# Comprehensive Hydrodynamic Investigation of Zebrafish Tail Beats in a Microfluidic Device with a Shape Memory Alloy

**DOI:** 10.3390/mi12010068

**Published:** 2021-01-09

**Authors:** Satishkumar Subendran, Chun-Wei Kang, Chia-Yuan Chen

**Affiliations:** Department of Mechanical Engineering, National Cheng Kung University, No. 1 University Road, Tainan 701, Taiwan; n16087046@mail.ncku.edu.tw (S.S.); n16081197@mail.ncku.edu.tw (C.-W.K.)

**Keywords:** zebrafish, shape memory alloy (SMA), microfluidics, tail beat, hydrodynamics

## Abstract

The zebrafish is acknowledged as a reliable species of choices for biomechanical-related investigations. The definite quantification of the hydrodynamic flow physics caused by behavioral patterns, particularly in the zebrafish tail beat, is critical for a comprehensive understanding of food toxicity in this species, and it can be further interpreted for possible human responses. The zebrafish’s body size and swimming speed place it in the intermediate flow regime, where both viscous and inertial forces play significant roles in the fluid–structure interaction. This pilot work highlighted the design and development of a novel microfluidic device coupled with a shape memory alloy (SMA) actuator to immobilize the zebrafish within the observation region for hydrodynamic quantification of the tail-beating behavioral responses, which may be induced by the overdose of food additive exposure. This study significantly examined behavioral patterns of the zebrafish in early developmental stages, which, in turn, generated vortex circulation. The presented findings on the behavioral responses of the zebrafish through the hydrodynamic analysis provided a golden protocol to assess the zebrafish as an animal model for new drug discovery and development.

## 1. Introduction

There is an urgent need for the advancement of new medication screening strategies dependent on in vivo approaches using several small animal models, such as *Caenorhabditis elegans*, *Drosophila melanogaster*, and *Danio rerio*. These animal models are gaining increasing interests as the screening tools for drug discovery due to their genetic agreeability, low cost, and culture conditions that are compatible with an enormous scope of screening [1,2]. Although several small animal models dependent on the traditional mammalian species have been developed to study human disease, the zebrafish is considered an ideal model for high-throughput studies in disease investigations. The zebrafish is much more complex than cultivated cells and other model organisms, for example, *Drosophila melanogaster*. In addition to this, toxicity experiments in the zebrafish are less expensive and time-consuming than those in rodents [2]. The zebrafish has become a mainstream research organism for toxicology studies as it possesses numerous advantages, for example, ease of husbandry, high fertility, small size, and rapid development [3,4]. It is vital to know that responses to external stimuli can be identified from early developmental stages of zebrafish larvae, as a wide range of behavioral responses are controlled by the brain [5,6,7].

Drug discovery requires screening a high number of medication possibilities for their adequacy, cytotoxicity, and conceivable symptoms. In vivo animal models are common for drug screening as there is an increasing amount of research related to resembling in vivo conditions within microfluidic systems [8]. Traditional approaches in the field of behavioral researches involve exposure of freely moving zebrafish larvae to chemicals/drugs in Petri dishes and multi-well plates. In addition, for microscopic imaging in desirable orientations of the zebrafish model organism, manual manipulation with forceps on agarose substrate or complete immobilization with anesthetics is needed [9,10,11]. This manual practice is not only time-consuming but also incorporates significant substantial and morphological damage, which can lead to adverse zebrafish conditions, and it is not suitable for easy quantification of subtle behavioral responses, thus calling for alternative rapid and easy-to-use techniques [4,12]. To date, most of the zebrafish-screening- and imaging-associated studies relied heavily on skilled researchers with repetitive and generally manual zebrafish orientation control activity to image the region of interests [13,14]. The above shortcomings have inspired the development of microfluidics as an ideal form of miniaturization technology for the screening of the zebrafish with precision in order to provide significantly analytical efficiency and high-throughput effectiveness with no loss of accuracy and reliability in reduced expense, time, and energy expenditure [15].

Flexible microfluidics has recently gained considerable interest, particularly in the miniaturization approaches in drug delivery for biomedical applications and wearable sensing technologies. To overcome the need for rigid supporting structures, there is a need for actuation technologies that can provide local strength and deformation, but without permanently reducing overall structural compliance. Shape memory alloy (SMA) technology has been used, as a microactuator, is capable of contracting when electrically activated. In general, an SMA wire’s actuating mechanism allows it to shrink when heated and return to its original length when cooled down to its original temperature. Thus, SMAs are also known as a memory metal, because they have the ability to return to their memorized shape after they are deformed [16]. The first SMA-wire-based microvalve for microfluidic chip applications was presented by Vyawahare et al. [17]. SMAs are actively used in many applications due to their shape memory effect and super elasticity capabilities [18]. This is because of their high-power density, large output displacements/forces and low operating voltages [19].

Rapid advances in science and technology have allowed the detection and characterization of possible toxicants. However, it is known that an animal’s exposure to chemicals may cause pain and additional distress. Hence, alternatives to animal testing are increasingly required for legal considerations, in addition to reducing both the time and cost of these studies. Although past toxicity studies have shown important impacts on adult zebrafish locomotion, a lack of high-throughput techniques for assessing zebrafish tail-beating motion has hampered a detailed understanding of the motor control modalities. There are several studies that have indicated the potential risk factors of cochineal red food additives on human health as well as in other model organisms [20,21]. The European Food Safety Authority, which intends to explicitly examine the toxicity of food coloring and provide information on food evidence, have issued the acceptable daily intake (ADI) limit for most food color additives, including cochineal red (ADI of 5 mg/kg bw/day) [22,23]. A novel microfluidic device coupled with an SMA actuator as a new experimental approach has been developed to immobilize zebrafish larvae. Thus, the approach of connecting a biological experiment with a flow visualization method to quantify the influence of cochineal red food additives on zebrafish larvae behavior with respect to tail beating is proposed and investigated.

## 2. Materials and Methods

### 2.1. Microfluidic Device Fabrication

**Figure 1:**
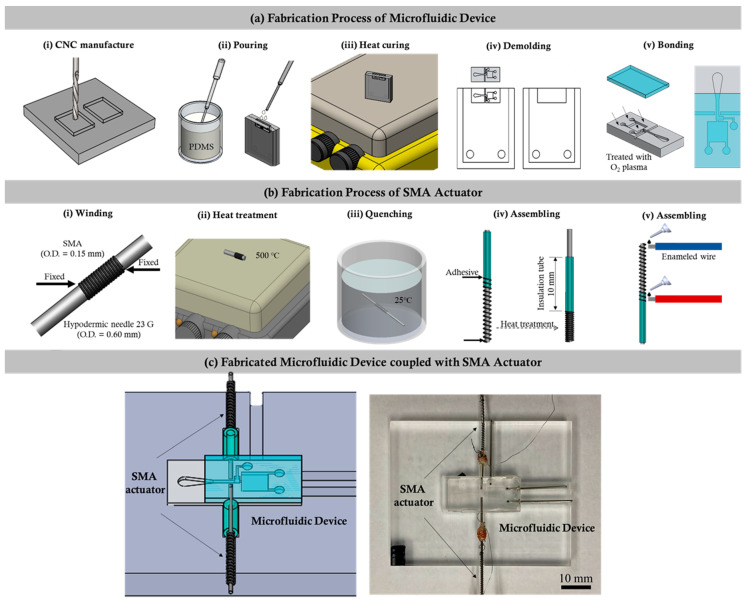
The fabrication process of the microfluidic and shape memory alloy (SMA) device. (**a**) i–v illustrates the various fabrication processes involved in the making of the proposed microfluidic device. (**b**) i–v illustrates the series of fabrication processes involved in the fabrication of the SMA device. (**c**) Illustration of the fabricated microfluidic device with the SMA integrated together.

The design details of the microfluidic device coupled with the SMA actuator used to quantify the instantaneous tail beating of the zebrafish are depicted in the Figure 1. The fabrication process of the microfluidic device consists of a series of computerized numerical control micromachining processes with the polydimethylsiloxane (PDMS) casting process. Firstly, the geometric design of the microfluidic device was inscribed on an acrylic substrate using a micromilling technique. The molds were affixed to fill up with the mixture of PDMS and curing agents weighing in a ratio of 10:1. In addition, the degassing process was carried out by following the curing process through hot plate baking at 95 °C for 48 h. Finally, the device was peeled from the parent mold [24,25,26], as shown in Figure 1a.

### 2.2. Fabrication of the Shape Memory Alloy (SMA) Actuator

In this work, an actuator was designed to fix the zebrafish in the observation region through SMA actuation. The flow chart illustrating the fabrication process of the SMA actuator is illustrated in Figure 1b. Initially, one end of a nickel–titanium (Ni–Ti) SMA guidewire (Flexinol, Dynalloy, Inc., Irvine, CA, USA) with a diameter of 0.60 mm was fixed on a 23 G hypodermic needle, and, then, the guidewire was wrapped along the needle without interspace between each wrap, forming a coil structure. After the coil reached a length of 2 mm, the guidewire was cut, and the other end was also fixed on the needle. Secondly, the coil along with the needle was heated with a hot plate at 500 °C for 10 min and then quenched in cold water. Once the SMA coil was fully cooled, the definition process of the original shape was finished. Thirdly, a small piece of borosilicate capillary tube with an inner and outer diameter of 0.55 and 1.4 mm, respectively, as well as a 24 G hypodermic needle with an outer diameter smaller than that of the SMA coil, was cut using a diamond dust rotary cutter and trimmed by sandpaper. To assemble the actuator, the SMA coil was gently stretched to an appropriate length, and its ends were glued to the hypodermic needle and borosilicate capillary tube with the help of polyurethane adhesive (Gorilla glue). Finally, the actuators were integrated into the microfluidic channel, as illustrated in the figure. The SMA coil in the actuator was designed to act as a spring that regained its preprogrammed shape and, thus, generated the force once its temperature exceeded a certain value. As depicted in Figure 1c, the borosilicate capillary part was attached to the microchannel that pushed the hypodermic needle towards the PDMS wall and compressed the wall during the SMA actuation. Once deactivated, the actuator returned back to its original position due to the elasticity of the compressed PDMS wall. In this work, the deformation of the SMA coil was controlled by applying electric currents with varying strengths.

### 2.3. Zebrafish Culture

Transgenic zebrafish larvae Tg (Cmlc2: eGFP/H2A: mCherry) were used, and their fertilized eggs were raised and cultured in edible additive solutions at a temperature ranging 28 °C ± 1 °C in a light: dark cycle of 14 h: 10 h. All experiments were performed under the relevant laws and institutional guidelines set by the National Cheng Kung University Institutional Animal Care and Use Committee (IACUC) with approval number: 106313.

### 2.4. Food Additive Preparation

In this experimentation, the cochineal red edible food additive was used. It was diluted from the 2% weight concentration of the original solution. For experimentation, 0.2‰ weight concentration of the cochineal red additive was brought into the zebrafish treatment in the microchannel. The zebrafish embryos of 6 h post fertilization (h.p.f.) were exposed to the daily refreshed embryo water containing food color additive for 6 days before further analysis and data collection. In addition, the control group was treated with fish water in E3 medium separately.

### 2.5. High-Speed Imaging of Zebrafish Tail Motion

Microparticle image velocimetry (μPIV) was used to provide a quantitative measure of how the cochineal red food additive can influence the cardiovascular functions of the zebrafish, specifically in terms of swimming behaviors. In order to minimize the harmful effect in which a required fluid tracking component can occur, the fluorescent particles were replaced by smashed egg yolk particles with better biocompatible properties. The cooked egg yolk weighing 0.04 g was mashed thoroughly in 1.5 mL of deionized (DI) water inside a conical tube. Furthermore, the diluted egg yolk mixture was vortexed for about 1–2 min until it appeared as a homogenous mixture. The prepared egg yolk particle solution was infused into the designed microfluidic device for the observation of the induced fluid flow pattern disturbed by zebrafish fin beating. Images were obtained using an optical microscope (BX60, Olympus Corp., Tokyo, Japan) and a high-speed camera (NX4-S2, IDT, Tallahassee, FL, USA) at a speed of 1000 frames per seconds (fps). The quantified flow fields were calculated by a particle image velocimetry (PIV) software package (Dynamic Studio, Dantec Dynamic A/S, Skovlunde, Denmark), and the results are presented as the calculated velocity vectors overlapped with the vorticity contour map. The first-pass PIV interrogation window was 64 × 64 pixels with 50% overlap for 10 iterations, and the second-pass was a 32 × 32 pixel interrogation size with 50% overlap for 10 iterations. Grid interpolation was used to increase the vector distribution and effectively reduce the bad vector and outliers.

### 2.6. Statistical Methods

To assess the impact of the cochineal red food additive on zebrafish cardiovascular responses, a statistical algorithm was used to analyze the data. The factors affecting the final heartbeat results included the food additive types and concentration together with the number of days after the fertilization of zebrafish eggs. The control group refers to the zebrafish without food additive treatment. For the comparison of the induced flows by tail beats, a two-tailed independent Student’s *t*-test was selected to compare the significant difference among the control group and the food coloring group.

## 3. Results

In the initial tests, the cochineal red (0.2‰) food additive was used for the assessment of the cardiovascular functionality in the transgenic zebrafish within the microfluidic device. It was noted that the average heart rate of the zebrafish 6 days post fertilization (6 d.p.f.) in the control group was measured at 218.78 ± 16.02 beats per minute (bpm), and that of the exposure to the cochineal red (0.2‰) was measured at 283.73 ± 30.54 bpm, which is significantly higher than that of the control group (*** *p* < 0.001). Furthermore, quantification of the tail-beating behavior with respect to the increased heartbeat rate of the zebrafish was investigated. In the current study, the zebrafish was positioned in the observation region within the fabricated microchannel coupled with the SMA actuator. The design details and the fabricated microfluidic device coupled with the SMA actuator for experimentation are shown in Figure 1. To test the influence of the fabricated microfluidic device on the control group (N = 10) zebrafish, the heart rate was measured before entering to the channel and after entering the channel. Interestingly, it was observed that the microfluidic device did not influence the heart rate of the zebrafish (See Supplementary Figure S1).

**Figure 2:**
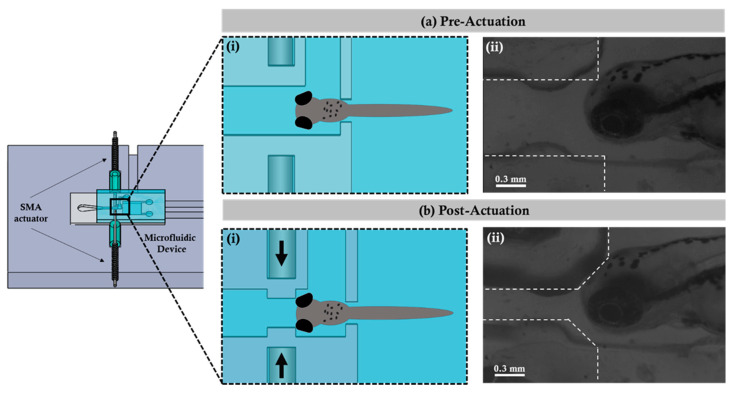
Illustration of the microfluidic device actuating operation using the SMA. (**a**) (**i**) and (**b**) (**i**) are the pictorial illustrations of the microfluidic device before and after the actuation process. (**a**) (**ii**) and (**b**) (**ii**) are the microphotographs captured before and after the actuation process.

To quantify the optimal values of displacement and the bending angle, as well as to identify the reliability, the miniaturized actuator was initially tested by actuating the single SMA; the distance between the microchannel wall was measured at 0.45 mm, and the overall change was calculated to be 10%. In addition, two SMA actuators were tested symmetrically to deform the wall; 0.2 mm was the observed distance between the microchannel wall, and the overall change was calculated as 46.9%. Thus, symmetrical actuation was employed to immobilize the zebrafish in the observation region in order to quantify the tail-beating behavior. Figure 2 illustrates the immobilizing mechanism using the SMA micro actuator coupled with the designed and fabricated microfluidic device. In the preactuation state, the zebrafish was transported and hydrodynamically fixed in the observation region within the microfluidic device. When the zebrafish was fixed in the observation region, the current was provided to the SMA, which worked via the mechanism of the thermal effect through the supplied current. The electrical current applied at the beginning was 1.8 A, and then it dropped to 1.5 A once the actuation occurred to prevent the device from overheating. The actuation was observed through a microscope during the experiments. At this time, the SMA returned to the shape which was previously set to achieve the effect of propulsion for squeezing the outer wall of the microchannel, thereby causing internal wall displacement. According to the experimentation, the inner wall shrinkage was achieved with a maximum displacement (i.e., minimum distance) of 0.204 ± 0.01 mm, and it finally reached the post-actuation state. After the post-actuation process, the zebrafish would not be able to move further and was immobilized in the observation region. Importantly, differences in the temperature during SMA actuation did not cause any sort of physiological damage to the zebrafish. The wall shrinkage percentage with change in time periods is graphically illustrated in the Supplementary Figure S2. The fabricated microfluidic system was capable of manipulating zebrafish larvae without using anesthetics or rigid gels; this enabled the further investigation of the hydrodynamic quantifications by correlating the behavioral changes with the instantaneous tail beating of the zebrafish larvae, which was not feasible in most existing strategies (See Supplementary Video S1).

**Figure 3:**
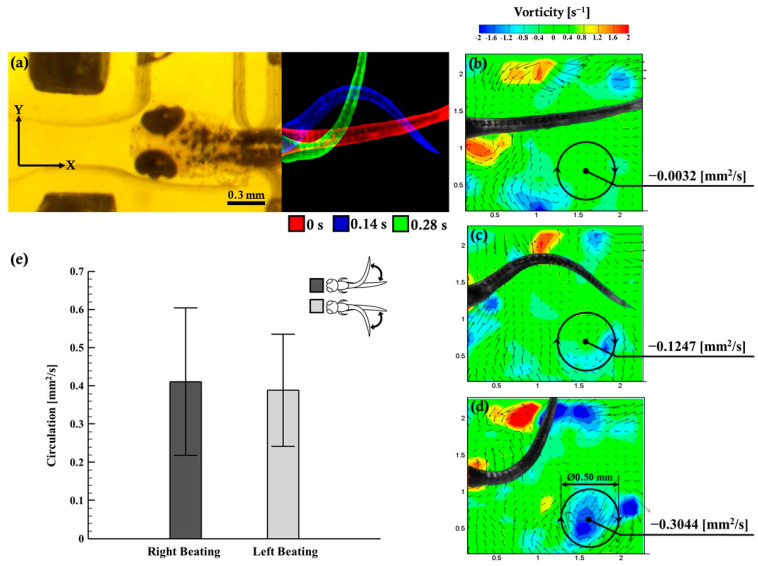
(**a**) The microphotograph of the zebrafish (6 d.p.f.) fixed in the observation region for tail quantification time. (**b**–**d**) The hydrodynamic quantificational measures of the zebrafish tail beating. The vorticity field (color map) is overlapped with the velocity vector field (black arrows) generated during tail beatings of zebrafish larvae (6 d.p.f.). (**e**) Instantaneous circulation formation due to the hydrodynamic right and left tail beating of zebrafish larvae (4, 5, and 6 d.p.f.) with effective test samples in each experimental group (N = 10).

Furthermore, hydrodynamic quantification of the generated vorticity was performed by zebrafish tail beating. Figure 3a depicts the trajectory of the zebrafish beating promptly where the zebrafish (6 d.p.f.) was fixed inside the observation region after the post-actuation process as discussed in Figure 2b. Figure 3b depicts the flow field overview at t = 0 s (beginning of the tail beat) which initiated the circulation. Figure 3c shows the position of the zebrafish body swing and the instantaneous flow field at t = 0.14 s; the circulation was measured at −0.1247 mm^2^/s. Similarly, Figure 3d depicts the instantaneous flow field at t = 0.28 s (the body swing reached 90°) and the attained maximum circulation of −0.3304 mm^2^/s. (the vortex ring was set as ø = 0.50 mm). It was observed that at the bottom right at t = 0.28 s, an obvious vortex ring was generated. As the zebrafish flexed its body into a “C” shape, it tended to draw its head and tail together by pushing and pulling the surrounding fluid environment and, thus, generating vortex circulation around the body into the flow. Figure 3e graphically illustrates the right and the left (burst and cruise) tail beating of the zebrafish while imparting the vorticity and generating the flow field. The circulation during right beating was measured at 0.4111 ± 0.1935 mm^2^/s, and that of left beating was measured at 0.3881 ± 0.1466 mm^2^/s. Previous studies have suggested that zebrafish larvae at various developmental stages produce different locomotory behavioral changes [27]. The current study focused on behavior changes in zebrafish larvae from 4 d.p.f. to 6 d.p.f., where the zebrafish locomotion was characterized by a burst-and-coast swimming style for fishes moved forward (burst) in one motion and gliding (coast) to a slow point, or stopping, from which they bursted forth again [28]. The current investigation employed the µPIV tool for precise visualizing of the flow physics close to the immobilized zebrafish tail beating region in order to gain a mechanistic understanding of its locomotion. Each tail beat cycle (locomotor cycle) was defined using three points, as illustrated in Figure 3b–d.

**Figure 4:**
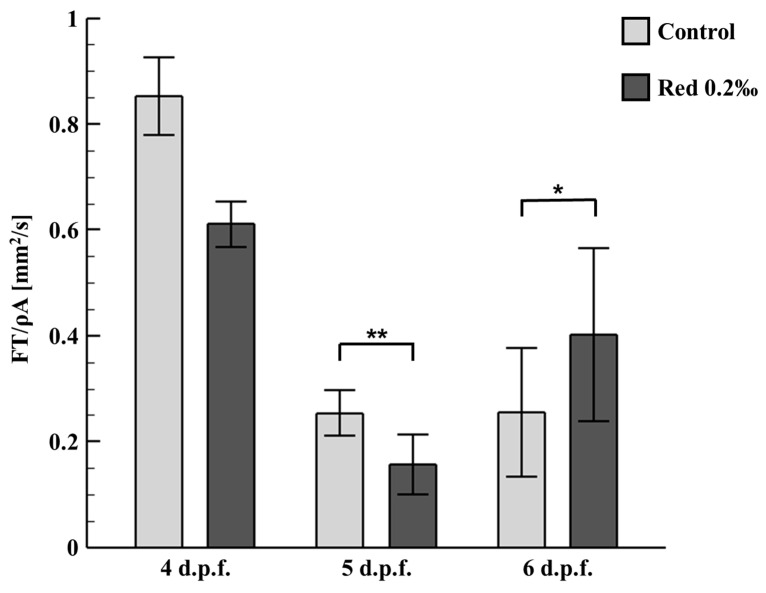
Effects of the cochineal red additive on tail-beating force of zebrafish larvae (4, 5, and 6 d.p.f.). Zebrafish larvae (5 and 6 d.p.f.) showed a transit change in the tail-beating force hydrodynamically with effective test samples in each experimental group. Significant differences between the control and the exposure groups are indicated by asterisks, (* *p* < 0.05) and (** *p* < 0.01) as compared with the control for 4, 5 d.p.f. (N = 10), and 6 d.p.f. (N = 30).

The startle and escape responses of the zebrafish have been previously studied using a variety of techniques [29,30]. Most of the previous investigations concluded that the startle response was interconnected to the neural interactions that are responsible for the escape behavior in fishes [31]. Startle response behaviors can be categorized into three distinct body-bending axial motions: the C-bend, withdrawal, and the S-bend. However, relatively little is known about the hydrodynamics of the escape responses of the zebrafish, given the fact that considering fluid flow patterns during escape is crucial for determining how body behavioral activity transmits power to the fluid media in order to identify the time course of power generation. Figure 4 illustrates the graphical representation of the instantaneous circulation comparison of the control group (N = 17) and the cochineal red additive group (N = 34). The average instantaneous circulation of the zebrafish exposed to the cochineal red group was measured at 0.3880 ± 0.1466 mm^2^/s, which is much higher than that of the control group. The significant difference between the control and cochineal red group (* *p* < 0.05) was obvious, because the cochineal red additive effects the behavioral and heart rate changes of the zebrafish larvae.

A locomotor force (F) experienced by the tail beating was calculated using Equation (1), where ρ is the water density, Γ is the circulation generated, A is the projected area of the vortex ring, and T is the time period of the tail beat. The change in fluid momentum caused due to the locomotory force of the zebrafish was quantified as a change in wake structure (i.e., circulation formation due to the tail beating). As a result, circulation was considered as a factor to evaluate the inferences generated by the zebrafish tail beating.

F = ρΓA/T,(1)

Figure 4 statistically illustrates the instantaneous circulation formation due to the hydrodynamic tail beating of zebrafish larvae (4, 5, and 6 d.p.f.) with effective test samples in each control group (N = 10). The average instantaneous circulation of the zebrafish (4 d.p.f.) exposed to cochineal red was measured at 0.6105 ± 0.0435 mm^2^/s, which is significantly higher than that of the zebrafish (5 and 6 d.p.f.). However, in the comparison between the zebrafish of 5 d.p.f. and 6 d.p.f., no major differences were observed. No major difference was observed in the zebrafish (4 d.p.f.) since the early stages of development; zebrafish larvae undergo predatory behavioral changes and become stable at later stages of development [32,33]. However, as per the results obtained in the present study, the zebrafish (5 d.p.f.) exposed to the cochineal red additive had a significantly lower tail-beating circulation of 0.1562 ± 0.0560 mm^2^/s as compared to that of the control group, which was measured at 0.2535 ± 0.0435 mm^2^/s. Secondly, in the case of the zebrafish (6 d.p.f.), the higher tail-beating circulation was measured at 0.4023 ± 0.1631 mm^2^/s which is significantly higher than that of the control group, which was measured at 0.2553 ± 0.1212 mm^2^/s.

## 4. Conclusions

The experimental study of zebrafish locomotion, with the integration of the latest technologies for visualizing fin and body movement and for quantifying hydrodynamic flow produced by the body and fins, has provided many insights in recent decades. Many of the interesting synchronized movements performed by larval zebrafish occurred instantaneously, and they are controlled by the neural circuits; thus, as a result, they can be investigated/analyzed by employing high-speed imaging strategies. The zebrafish escaping responses were significantly important behaviors, with comprehensive investigation on both the neural regulation and the biomechanics of the performance of the startle response. In the present study, the zebrafish behavioral response test was performed within the fabricated novel microfluidic paradigm coupled with the SMA actuator. The insights from the investigation were applied to perform a behavioral study of zebrafish exposed to a cochineal red additive and that of a control group. It is suggested that a more detailed assessment of the effects on activities required the evaluation and integration of multiple parameters of locomotor and kinematic activities. In particular, the behavioral response of the zebrafish was seen as a useful and appropriate endpoint to diagnose neuroactive substances. Future research should expand the zebrafish model organism to address behavioral neuroscience problems, an undertaking that involves accurate and efficient behavioral investigation methodologies.

## Data Availability

The presented data are available upon request.

## Supplementary Materials

The following are available online at https://www.mdpi.com/2072-666X/12/1/68/s1, Figure S1: Graphical representation data—influence of a microchannel on zebrafish larvae; Figure S2: Illustration of the microchannel’s wall distance before and after the actuation process using the SMA micro actuator; Video S1: Overall experimental working process with a description.
