# Supplementary material for: Comprehensive Hydrodynamic Investigation of Zebrafish Tail Beats in a Microfluidic Device with a Shape Memory Alloy
Source: Micromachines (Basel). 2021 Jan 9;12(1):68. doi: 10.3390/mi12010068 (PMC7827268; doi:10.3390/mi12010068)
Supplement: Supplementary file 1 [file micromachines-12-00068-s001.zip › Electronic Supplementary-1-7-2021.docx]

**Electronic** **Supplementary Information (ESI) for**

**Comprehensive Hydrodynamic Investigation of Zebrafish Tail Beats in a Microfluidic Device with a Shape Memory Alloy**

By

Satishkumar Subendran^1,†^, Chun-Wei Kang^1,†^, and Chia-Yuan Chen^1,*^

^1^Department of Mechanical Engineering, National Cheng Kung University, No. 1 University Road, Tainan, 701 Taiwan

†Equal contribution

*****Correspondence: chiayuac@mail.ncku.edu.tw; Tel.: +886-2757575-62169

*
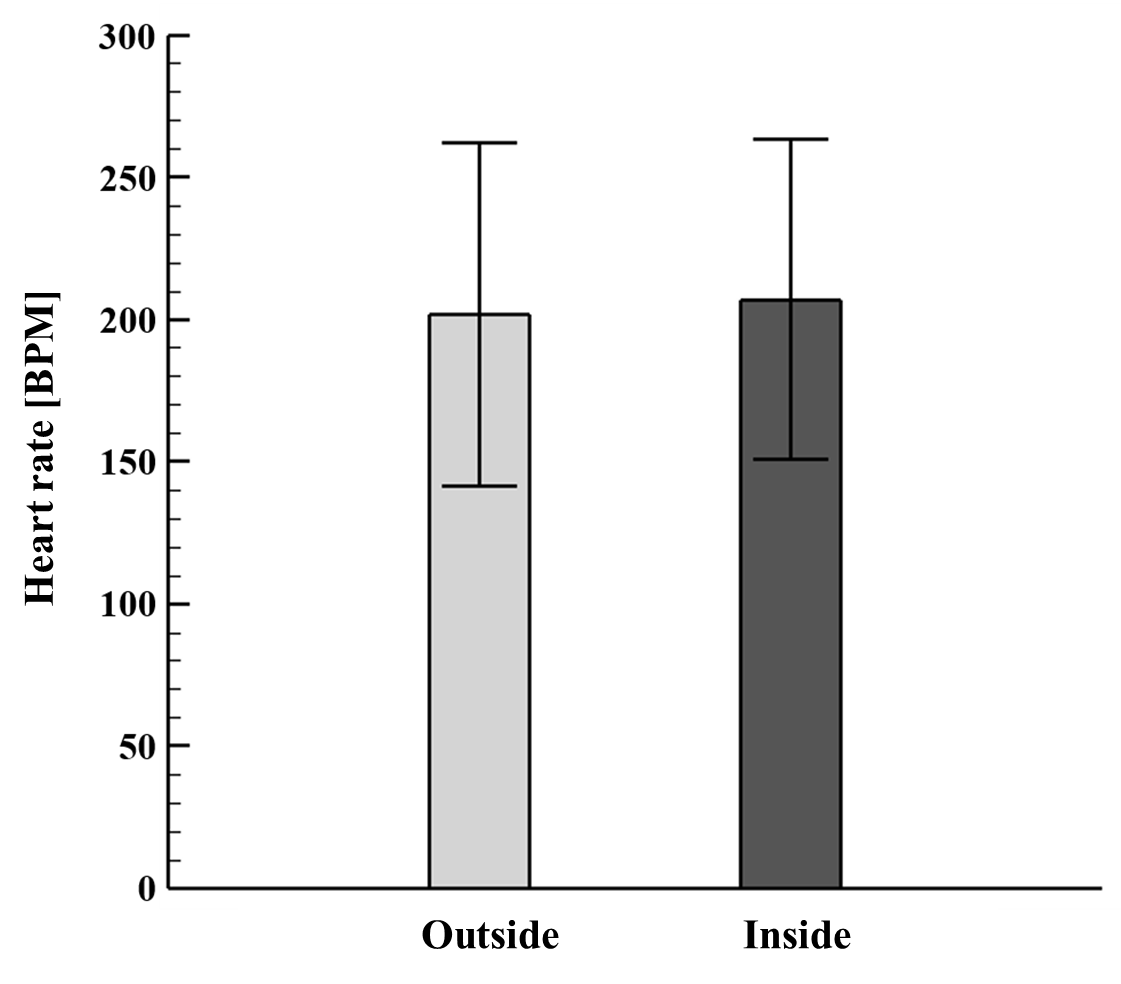
*

**Fig. S1.** The influence of the microchannel on zebrafish larvae was tested in this study. Heart rate data were compared between the outside and inside microchannels (outside & inside groups N=8). There is no significant difference between two groups by the statistic paired-sample T-test.

*
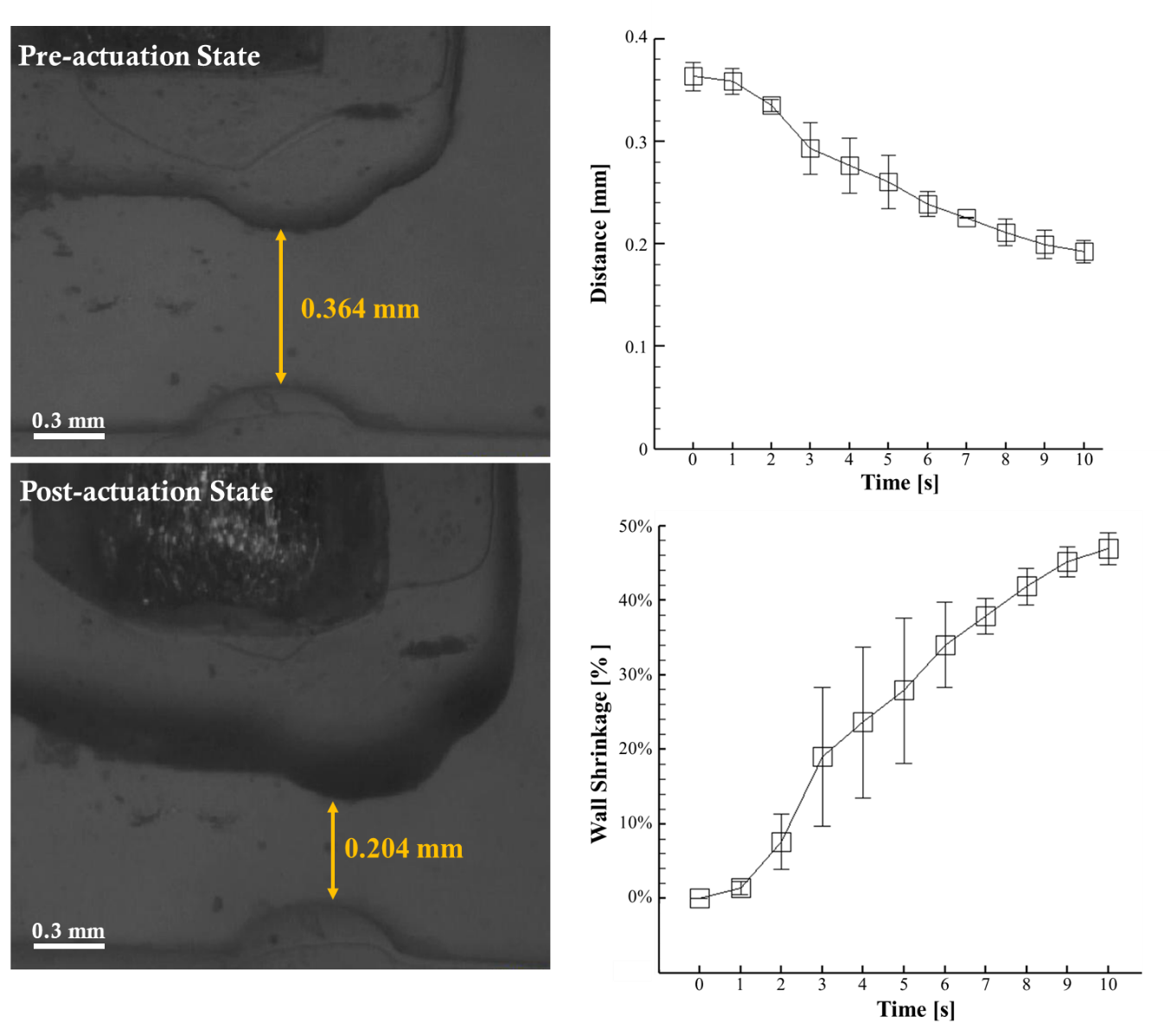
*

**Fig. S2.** Illustration of the microchanne wall distance before and after the actuation process using the SMA micro actuator. The microchannel wall reached to its minimum distance which was measured 0.204 ± 0.01 mm in a time period of 10 s. Additionally, the maximum wall shrinkage percentage was 46.9 ± 2.1 %.

**Supplementary Video S1 -** The experimental description of the proposed work.
